# Integrated metabolite profiling and transcriptome analysis unraveling mechanism of RC catabolism in *Paenarthrobacter ilicis* CR5301

**DOI:** 10.3389/fmicb.2023.1180388

**Published:** 2023-04-27

**Authors:** Hongfei Li, Daqing Sun, Longkui Cao, Baohui Wang

**Affiliations:** ^1^College of Chemistry and Chemical Engineering, Northeast Petroleum University, Daqing, China; ^2^National Coarse Cereals Engineering Research Center, Heilongjiang Bayi Agricultural University, Daqing, China

**Keywords:** rebaudioside C, transcriptome, β-glucosidase, *Paenarthrobacter ilicis* CR5301, catabolism, mechanism

## Abstract

Steviol glycosides are ideal sweeteners that are widely used in food, medicine, and cosmetics. Rebaudioside C (RC) is considered to be the third most abundant steviol glycoside, which has a bitter aftertaste that limits its application. Hydrolysis of RC to generate other bioactive steviol glycosides is an effective way to promote its additional utilization. In our previous study, a bacterium *Paenarthrobacter ilicis* CR5301 was isolated and identified for hydrolyzing RC with high efficiency. Herein, the expression profiles of *P. ilicis* CR5301 in the deletion and presence of RC were investigated by RNA-seq. The RC metabolites were identified by high-performance liquid chromatography and ultra-performance liquid chromatography-triple-time of flight mass spectrometry. Novel results were discovered in four aspects of research. First, the identification of metabolites revealed that four metabolites, namely, dulcoside A, dulcoside B, dulcoside A1, and steviol, were produced during RC metabolism. Second, RNA-seq analyses unraveled that 105 genes of *P. ilicis* CR5301 were significantly differentially expressed, and 7 pathways were significantly enriched. Third, independent RT-qPCR verified the accuracy and reliability of the RNA-seq results. Finally, a complete catabolic model of RC in *P. ilicis* CR5301 was proposed, and key genes were indicated in the RC catabolic metabolism by combining them with literature and sequence alignments. This study comprehensively unraveled the genes and pathways of RC catabolism in *P. ilicis* CR5301 at the transcriptional and metabolic levels. It provided new insights and evidence for understanding the mechanism of RC catabolism in bacteria. Key candidate genes may potentially contribute to the RC hydrolysis and preparation of other functional steviol glycosides in the future.

## 1. Introduction

Steviol glycosides, a low-calorie, high-sweetness sweetener extracted from the leaves of *Stevia rebaudiana* Bertoni, are ideal substitutes for sucrose (Savita et al., 2004; Huang et al., 2010). In addition to their high sweetness, steviol glycosides have been suggested to exert beneficial effects on human health, including noncariogenic, anti-hyperglycemic, anti-hypertensive, anti-cancer, anti-inflammatory, and anti-microbial activities, and are widely used in food, cosmetics, medicine, and other industries (Gardana et al., 2003; Lemus-Mondaca et al., 2012). There are many components of steviol glycosides, mainly including stevioside (ST), rebaudioside A (RA), rebaudioside C (RC), and some lower concentrations of dulcoside A (Duc A), rubusoside, rebaudioside B, steviolbioside, and other very minor amounts of steviol glycosides such as steviol (STE) (Gerwig et al., 2016).

Rebaudioside C is the third most abundant type of steviol glycoside (Hanson, 2016). It has a low sweetness and a bitter aftertaste that affect the taste of food and beverages. Thus, its application is limited (Yang et al., 2019). Previous studies have found that many glycosylation-modified products of all steviol glycosides have better sweetness and taste profiles (Hellfritsch et al., 2012; Wan, 2012). Only one study, specifically on the modification of RC, modified RC to a variety of new RC derivatives (α-1 → 6-glucosyl RC) using commercial glycosyltransferases and sucrose as a glucose donor (Yang et al., 2019). These new RC derivatives have increased sweetness, decreased bitterness, and enhanced solubility in water (Yang et al., 2019). However, enzyme modification also has some drawbacks, such as the difficult control of the amount of glycosyl group transfer, complex product composition, and tedious and expensive purification procedures, which greatly hinder the application of enzyme modification to RC (Wan, 2012).

Besides enzyme modification to debitter RC, the hydrolysis of the linkages of sugar residues at the C13 and C19 side chains of RC to produce other bioactive steviol glycosides is also an effective way to expand the utilization of RC. Currently, it has been found that the microorganisms capable of hydrolyzing RC were human intestinal microflora (Koyama et al., 2003), *Aspergillus aculeatus* (Ma et al., 2014), *Penicillium pinophilum* (Ma, 2014), and *Microbacterium barkeri* (Jiang et al., 2020). The human intestinal microflora is a mixture that can metabolize steviol glycosides such as ST, RA, and RC to STE. Meanwhile, the metabolic pathways of ST, α-monoglucosylstevioside, RA, and α-monoglucosylrebaudioside A in human intestinal microflora were proposed. The final product of these metabolic pathways was STE, but the metabolic pathway of RC was not mentioned (Koyama et al., 2003). Ma et al. found that the enzyme solution of *A. aculeatus* ZJ and *P. pinophilum* ZS2 could hydrolyze RC to STE (Ma, 2014; Ma et al., 2014). Additionally, the product of hydrolysis of RC using the mycelium of *A. aculeatus* ZJ was rubusoside rather than STE (Ma, 2014). Jiang et al. studied the conversion of steviol glycosides by *M. barkeri* XJ and found that ST, RA, and RC were completely hydrolyzed to STE at 24 h, 84 h, and 144 h, respectively. Furthermore, a deglycosylation pathway of steviol glycosides in *M. barker*i XJ was obtained. Due to the complex composition of the substrate steviol glycosides, it was uncertain which products in this deglycosylation pathway were derived from the metabolism of RC (Jiang et al., 2020). Microorganisms can hydrolyze RC due to the function of their enzymes. The hydrolysis of RC to STE and rubusoside by *A. aculeatus* ZJ indicates that more than one enzyme may be active at RC hydrolysis. Although human intestinal microflora, *A. aculeatus* ZJ, *P. pinophilum* ZS2, and *M. barker*i XJ, can hydrolyze RC to the same product, STE, the enzymes involved may be different. The above research demonstrated that the key genes and enzymes that play the function of RC hydrolysis have not been involved in these microorganisms. Therefore, it is necessary to further explore the specific enzymes in the strains with the RC hydrolyzing activity for the preparation of the particular steviol glycoside. To the best of our knowledge, the metabolic pathways and catabolic mechanisms of RC in microorganisms have also not been reported.

In this study, the transcription profiles of *Paenarthrobacter ilicis* CR5301 (Li et al., 2022a,b) from my laboratory, which can efficiently hydrolyze RC to STE, were determined by RNA-seq. and the metabolites of RC were detected and identified by high-performance liquid chromatography (HPLC) and ultra-performance liquid chromatography-triple-time of flight mass spectrometry (UPLC-Triple-TOF/MS). The aim of this study is to contribute a theoretical and experimental basis for elucidating the catabolic mechanism of RC at the transcriptional and metabolic levels and provide key candidate genes for the application of RC hydrolysis and the preparation of other functional steviol glycosides in the future.

## 2. Materials and methods

### 2.1. Strain, medium, and culture conditions

*Paenarthrobacter ilicis* CR5301 was isolated from *Stevia* planting soil and deposited at the China Center for Type Culture Collection (CCTCC NO.: M2021851). The activation medium and the control group medium were Czapek-Dox broth medium (NaNO_3_ 0.3%, KCl 0.05%, K_2_HPO_4_ 0.1%, FeSO_4_ 0.001%, MgSO_4_·7H_2_O 0.05%, and sucrose 3%), and the treatment group medium was supplemented with 2% RC standard in the control group medium. The CR5301 was incubated at 28°C for 24 h in the activation medium for the subsequent fermentation experiment.

### 2.2. RC fermentation trial

The CR5301 was activated for two generations, and the cultures were inoculated in the media of the control and treatment groups at 5% inoculum and cultivated at 28°C and 270 rpm. Samples were obtained from the control and treatment groups at intervals. Part of each sample was centrifuged at 4°C and 6,000 rpm for 15 min, and the cells collected by centrifugation were frozen immediately in liquid nitrogen for 5 min and were then stored at −80°C for RNA-seq. Then, the other part of the samples in the treatment group were boiled for 10 min, centrifuged for 5 min, and the supernatant were filtered by 0.22-μm filter membranes; then, concentrations of metabolites were determined by HPLC.

### 2.3. Metabolite identification and concentration assay

The concentrations of metabolites were determined by HPLC. Based on the HPLC method, the identification of metabolites was combined with UPLC-Triple-TOF/MS, and detailed contents were carried out according to the literature (Li et al., 2022b).

The RC hydrolysis rate per hour and the production efficiency of STE were calculated as follows:


$$\mathrm{RC}\;\mathrm{hydrolysis rate}\;\mathrm{per}\;\mathrm{hour}\;\left(\%/h\right)=\frac{C_{0}V_{0}-C_{t}V_{t}}{C_{0}V_{0}h}\times 100$$

$$\mathrm{STE}\;\mathrm{production efficiency}\;\left(g/h\right)=\frac{C1_{t}V_{t}M_{STE}}{h}$$

where *C_0_* is the initial RC concentration (mol/L), *C_t_* is the real-time RC concentration in the reaction mixture (mol/L), *V_0_* is the initial solution volume (L), *V_t_* is the real-time solution volume (L), and *h* is hydrolysis time. *C*1*_t_* is the real-time STE concentration in the reaction mixture (mol/L), and *M_STE_* is the molar mass of STE.

### 2.4. RNA-seq and data analysis

#### 2.4.1. Total RNA sample preparation

Both the control and treatment groups contained 3 biological replicates. Total RNA from 6 samples was extracted by TRIzol^®^ reagent according to the instructions (Invitrogen, United States), and genomic DNA was removed using DNase I (TaKaRa, JP). Then, the quality of RNA was measured by 2100 Bioanalyzer (Agilent, United States), and ND-2000 (NanoDrop Technologies, United States) was used for RNA quantification. High-quality RNA samples were used to construct the sequencing libraries.

#### 2.4.2. RNA-seq

The rRNA was removed from total RNA samples using Ribo-Zero™ Magnetic Kit (Epicenter, United States). The construction of the 6 transcriptome libraries and RNA-seq were performed by Majorbio Biotech Co. Ltd. (Shanghai, China), and the Illumina NovaSeq 6000 platform was used for RNA-seq. The reads data generated by RNA-seq can be accessed (SRA: SRP415665).

#### 2.4.3. Bioinformatics analysis

Filtered clean reads were mapped against predicted transcripts from the *P. ilicis* CR5301 genome using Bowtie. The gene expression levels were calculated using RSEM 1.3.1. The abundance of each transcript was calculated using transcripts per million reads (TPM). Genes with |log2 foldchange| ≥1 and an adjusted *p*-value ≤0.05 were considered to be significantly differentially expressed (SDE) genes. The organization of the operon was predicted by Rockhopper software. SignalP-6.0 and TMHMM-2.0 software were used to predict the signal peptide and transmembrane domains in proteins, respectively. Multi-sequence alignment was performed by the MEGA7.0.26 software.

### 2.5. Quantitative PCR verification

The 15 randomly selected genes were separately examined using reverse transcription quantitative PCR (RT-qPCR). Total RNA was isolated as described above and reverse transcribed using a PrimeScript RT reagent kit (TaKaRa, JP) according to the manufacturer’s instructions. The amplifications were performed using specific primers (Supplementary Table S1) and ChamQ SYBR Color qPCR Master Mix (Vazyme, China) with a 7300 real-time PCR system (ABI, United States). Gene expression was normalized by the 2^-(ΔΔCt)^ method, and the 16S ribosomal gene of strain CR5301 was used as the normalized standard.

### 2.6. Statistical analysis

All tests were performed in triplicate. The data from replicate experiments were represented as the means ± standard deviations. The one-way ANOVA was used to determine the significance of data differences between groups by GraphPad Prism 8.0.1 software.

## 3. Results

### 3.1. Fermentation experiment

**Figure 1:**
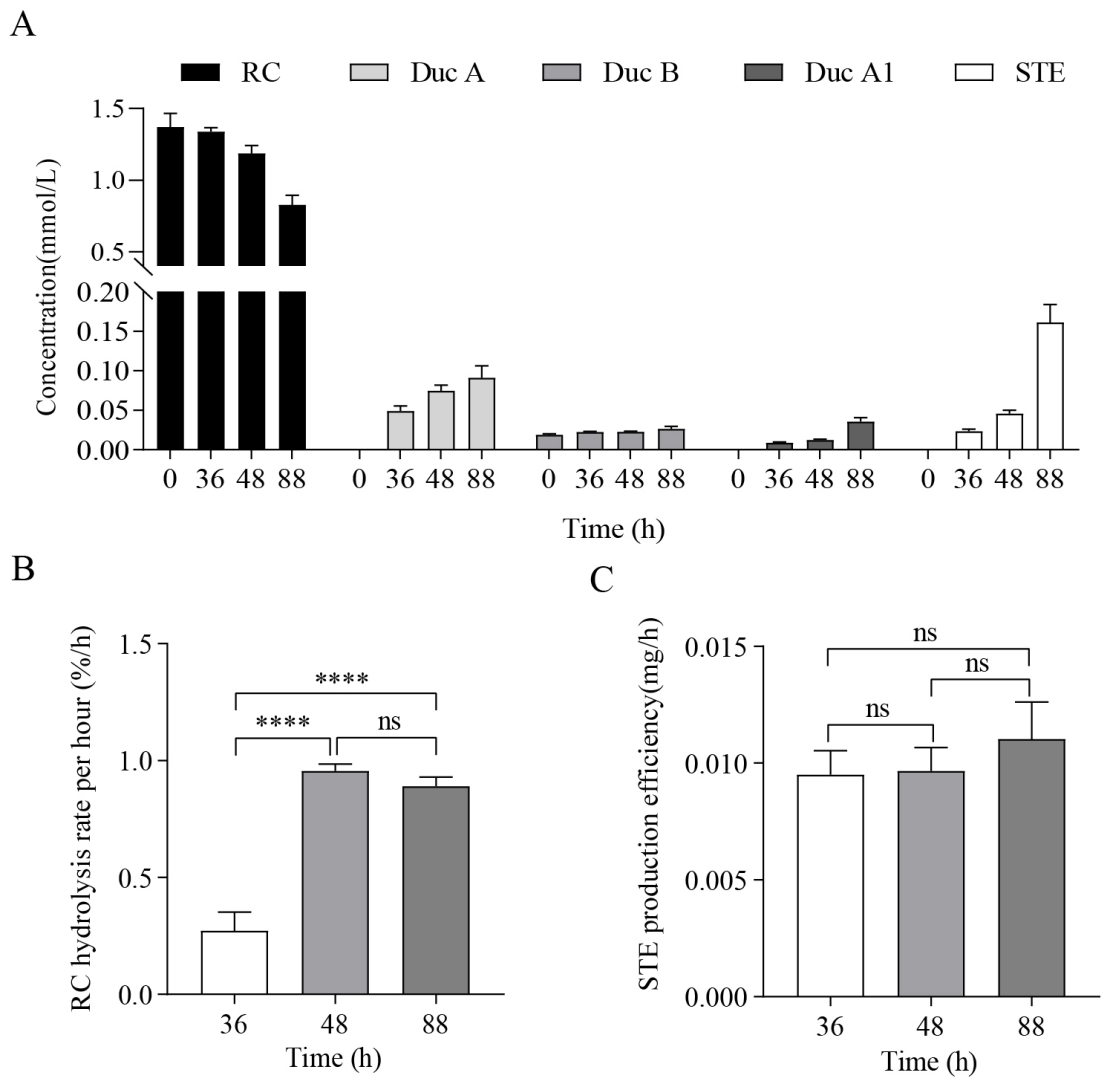
RC fermentation by *Paenarthrobacter ilicis* CR5301. **(A)** Concentration changes in RC and metabolites in the samples from the treatment groups at different times, **(B)** RC hydrolysis rate per hour at different times, and **(C)** STE production efficiency at different times. [**** *p* < 0.0001, NS: not significant (*p* > 0.05)].

The results of RC fermentation are shown in Figure 1. Figure 1A shows that, as fermentation time increases, the concentration of RC in the medium of the treatment group gradually decreases. Moreover, four new metabolites, namely, Duc A, dulcoside B (Duc B), dulcoside A1 (Duc A1), and STE, were detected. The concentration of STE was the highest among the four metabolites at 88 h. Previous studies have proven that STE was the final product of the metabolism of RC by CR5301 (Li et al., 2022b); therefore, STE accumulated the fastest among the four metabolites. Figures 1B,C show the RC hydrolysis rate per hour and STE production efficiency at different times; these two indicators were used to select RNA-seq samples. Figure 1B shows that the RC hydrolysis rate per hour at 48 h and 88 h was not significant, but they were significant compared with that at 36 h. Figure 1C shows that STE production efficiency at 36 h, 48 h, and 88 h was not significant to each other but showed an upward trend. According to the RC hydrolysis rate per hour and STE production efficiency, 48 h–88 h was the stage of high enzyme activity during the RC metabolism in CR5301; however, these two indicators at 48 h and 88 h were not significant, respectively. Therefore, both 48 h and 88 h samples can be used for RNA-seq. In this study, 88 h was selected.

### 3.2. Metabolite identification

**Figure 2:**
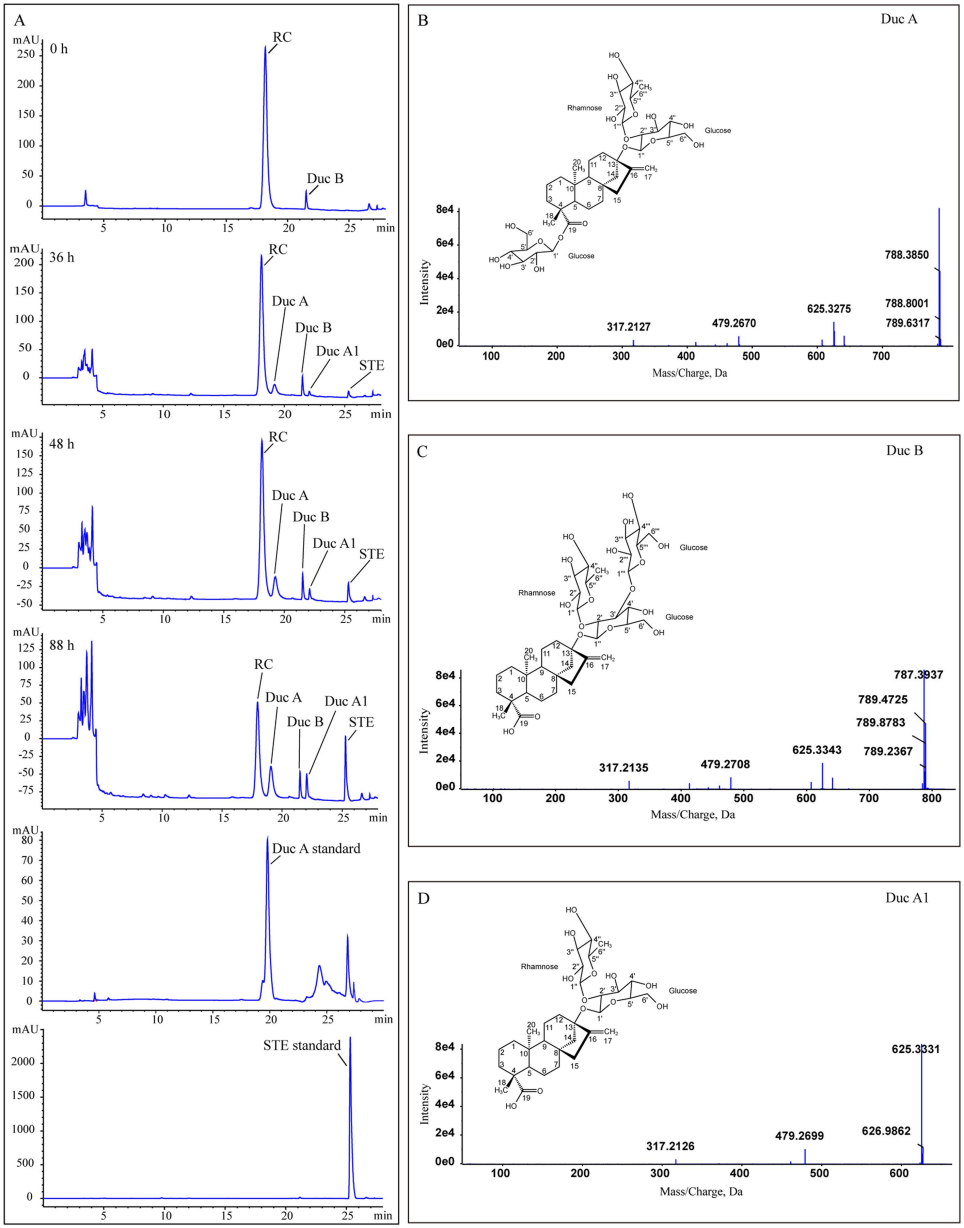
Identification of RC metabolites. **(A)** HPLC chromatograms of Duc A, SET standards, and samples from the treatment group at different times, **(B–D)** the MS/MS spectra and structures of Duc A, Duc B, and Duc A1, respectively.

Figure 2A shows that, as fermentation time increases, RC in the solution gradually decreases and is hydrolyzed to four metabolites. The metabolites were identified by UPLC-Triple-TOF/MS and HPLC methods. The formulas, sugar units at both C13 and C19 sites, and the fragment ions of the four metabolites are listed in Table 1.

**Table 1:** Formula and MS fragmentation of metabolites.

Metabolites No.	C13 portion	C19 portion	Formula	[M-H]^−^(*m/z*)	Major fragment ions(*m/z*)	Monosaccharide composition
Metabolite 1 (Duc A)	Rhaα(1–2)Glcβ1−	Glcβ1−	C_38_H_60_O_17_	787.3741	625.3275	Glc(2),Rha(1)
					479.2670	
					317.2127	
Metabolite 2 (Duc B)	Rhaα(1–2)[Glcβ(1–3)]Glcβ1−	H	C_38_H_60_O_17_	787.3733	625.3343	Glc(2),Rha(1)
					479.2708	
					317.2135	
Metabolite 3 (Duc A1)	Rhaα(1–2)Glcβ1−	H	C_32_H_50_O_12_	625.3209	479.2699	Glc(1),Rha(1)
					317.2126	
Metabolite 4 (STE)	H	H	C_20_H_30_O_3_	317.2120	−	−

**Metabolite 1.** Figure 2B shows the MS/MS spectrum of metabolite 1. [M-H]^−^ was m/z 787.3741, suggesting a molecular formula C_38_H_60_O_17_. According to this MS/MS spectrum, three major fragment ion peaks appeared, namely, m/z 625[M-H-162], 479[M-H-162-146], and 317[M-H-162-146-162], which indicated that two glucose units and a rhamnose unit existed in the structure of metabolite 1. Combined with the search results of the SciFinder and Reaxy databases and the HPLC chromatogram of the Duc A standard (Figure 2A), metabolite 1 was presumed to be Duc A.

**Metabolite 2.** Figure 2C shows the MS/MS spectrum of metabolite 2. [M-H]^−^ was m/z 787.3733, suggesting a molecular formula C_38_H_60_O_17_. Although the molecular formula of metabolite 2 was identical to that of metabolite 1, the retention time of the HPLC chromatogram of the two metabolites was different. Compared with the retention time of the Duc A standard, the possibility that metabolite 2 was Duc A could be excluded. According to this MS/MS spectrum, three major fragment ion peaks appeared, namely, m/z 625 [M-H-162], 479 [M-H-162-146], and 317 [M-H-162-146-162], which indicated that two glucose units and a rhamnose unit existed. According to the search results of the SciFinder and Reaxy databases, metabolite 2 was presumed to be Duc B.

**Metabolite 3.** Figure 2D shows the MS/MS spectrum of metabolite 3. [M-H]^−^ was m/z 625.3209, suggesting a molecular formula C_32_H_50_O_12_. According to this MS/MS spectrum, two major fragment ion peaks appeared, namely, m/z 479 [M-H-146] and 317 [M-H-146-162], which indicated that a glucose unit and a rhamnose unit existed. According to the search results of the SciFinder and Reaxy databases, metabolite 3 was presumed to be Duc A1.

**Metabolite 4.** [M-H]^−^ was m/z 317.2120, suggesting a molecular formula C_20_H_30_O_3_ (Table 1). Additionally, combined with the retention time of the HPLC chromatogram of the STE standard, metabolite 4 was presumed to be STE.

**Figure 3:**
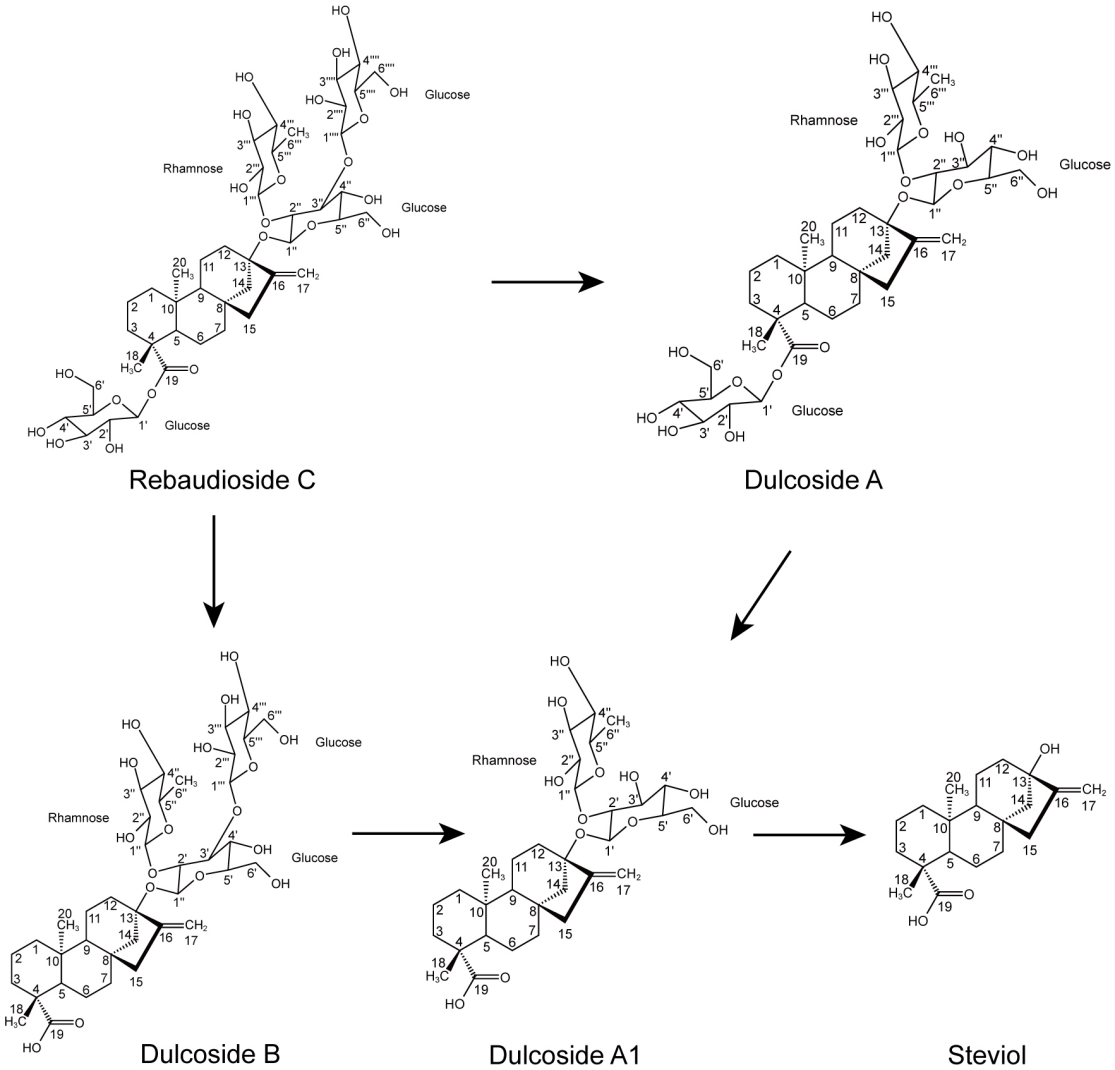
Proposed hydrolysis pathways of RC in *P. ilicis* CR5301.

According to the results of the RC fermentation experiment and the identification of four metabolites, the process of RC metabolism in *P. ilicis* CR5301 was mainly the hydrolysis process of glycosyl side chains of RC. Two hydrolysis pathways were predicted (Figure 3), namely, RC-Duc A-Duc A1-STE and RC-Duc B-Duc A1-STE.

### 3.3. Overview of the results of RNA sequencing

**Figure 4:**
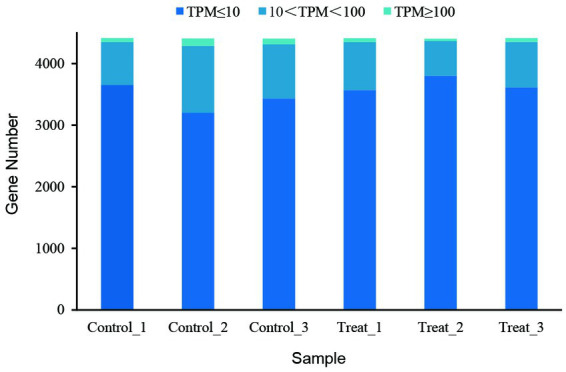
Gene expression abundance of the RNA-seq samples.

**Figure 5:**
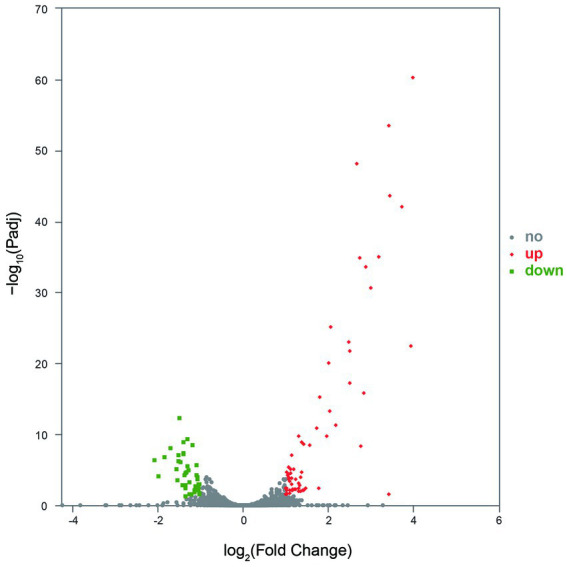
The SDE genes between the control and treatment groups. No indicates the gene with significantly non-regulated expression and abs(FoldChange) <1 or Padj >0.05. Up indicates the gene with significantly upregulated expression, log_2_FoldChange ≥ 1, and Padj ≤0.05. Down indicates the gene with significantly downregulated expression, log_2_FoldChange ≤ −1, and Padj ≤0.05.

Gene expression analysis showed that more than 4,400 genes were transcribed in each sample. Compared with the control group, the number of genes with low expression (TPM ≤ 10) was more in the treatment group, and the number of genes with medium expression (10 < TPM < 100) and high expression (TPM ≥ 100) was less (Figure 4). An analysis of significantly differentially expressed (SDE) genes showed 105 SDE genes (Supplementary Table S2), with 65 genes significantly upregulated and 40 genes significantly downregulated (Figure 5).

**Figure 6:**
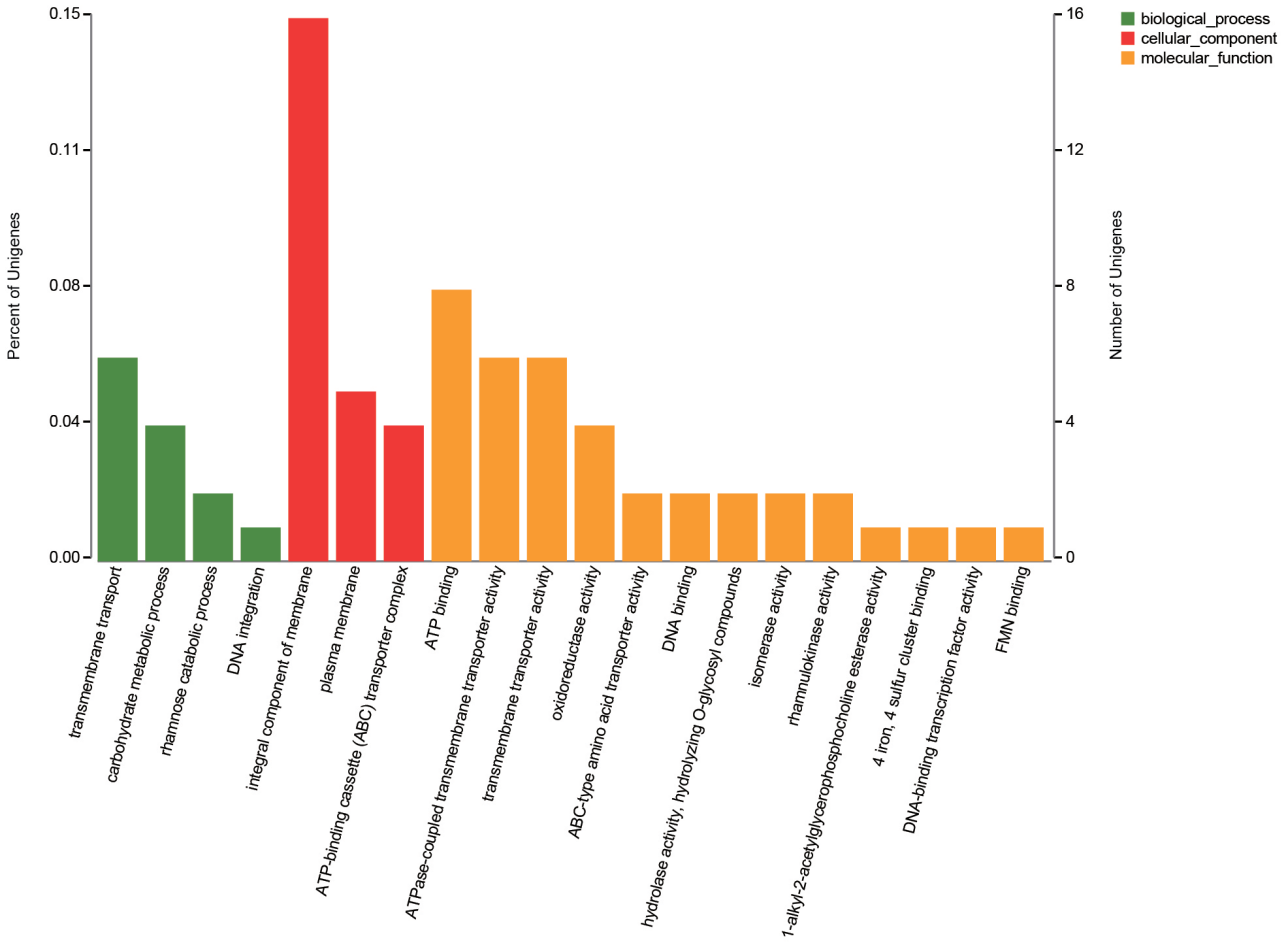
The GO annotation classification of the SDE genes.

**Figure 7:**
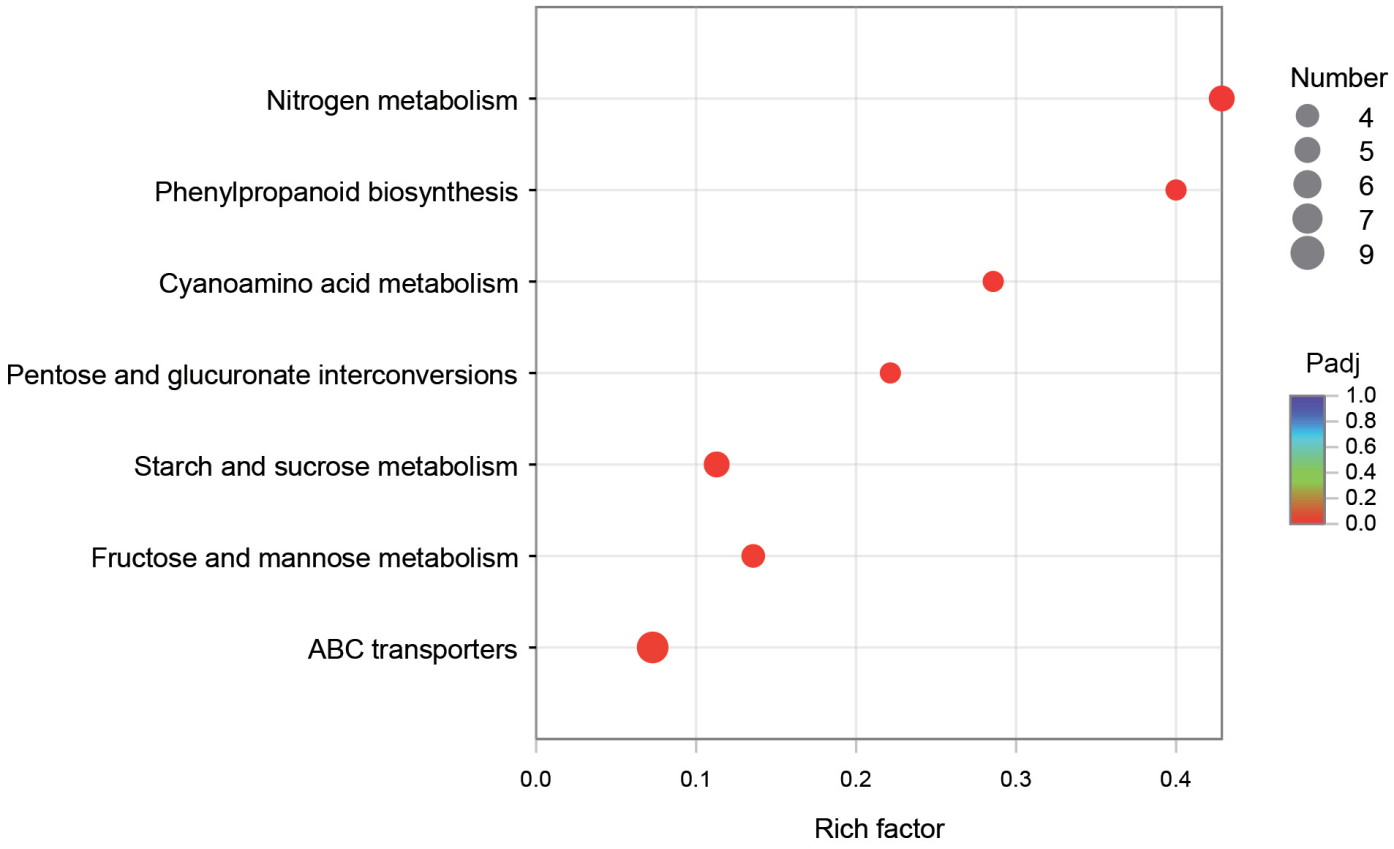
The pathway enrichment of the SDE genes.

The GO annotation classification of SDE genes showed that 12 terms were significantly enriched in the functional classification of “biological process” genes, mainly involving carbohydrate metabolism genes (8 terms), localization genes (2 terms), and transport genes (2 terms); 7 terms of “cell components” were significantly enriched, all of which were related to the genes of the membrane transporter complex; and 17 terms of “molecular function” were significantly enriched, mainly involving membrane transporters (13 terms) and carbohydrate hydrolysis (4 terms) (Figure 6). Pathway enrichment analysis of SDE genes showed that 7 pathways were significantly enriched (Padj≤0.05) (Figure 7).

### 3.4. SDE genes in the RC metabolism

Due to structural differences between RC and the four metabolites, partial or total hydrolysis of one glucosyl residue at the C19-carboxyl group and trisaccharide residue (two glucosyl residues and one rhamnosyl residue) at the C13-hydroxyl group can be achieved to metabolize RC into Duc A, Duc B, Duc A1, and STE. In other words, it is necessary to hydrolyze the β-glucosyl ester linkage at C19, two β-glucosidic linkages, and one α-rhamnosidic linkage at C13. Therefore, genes associated with the expression of enzymes with the hydrolytic activity of these glycosidic linkages are likely to be significantly upregulated after the addition of RC.

**Figure 8:**
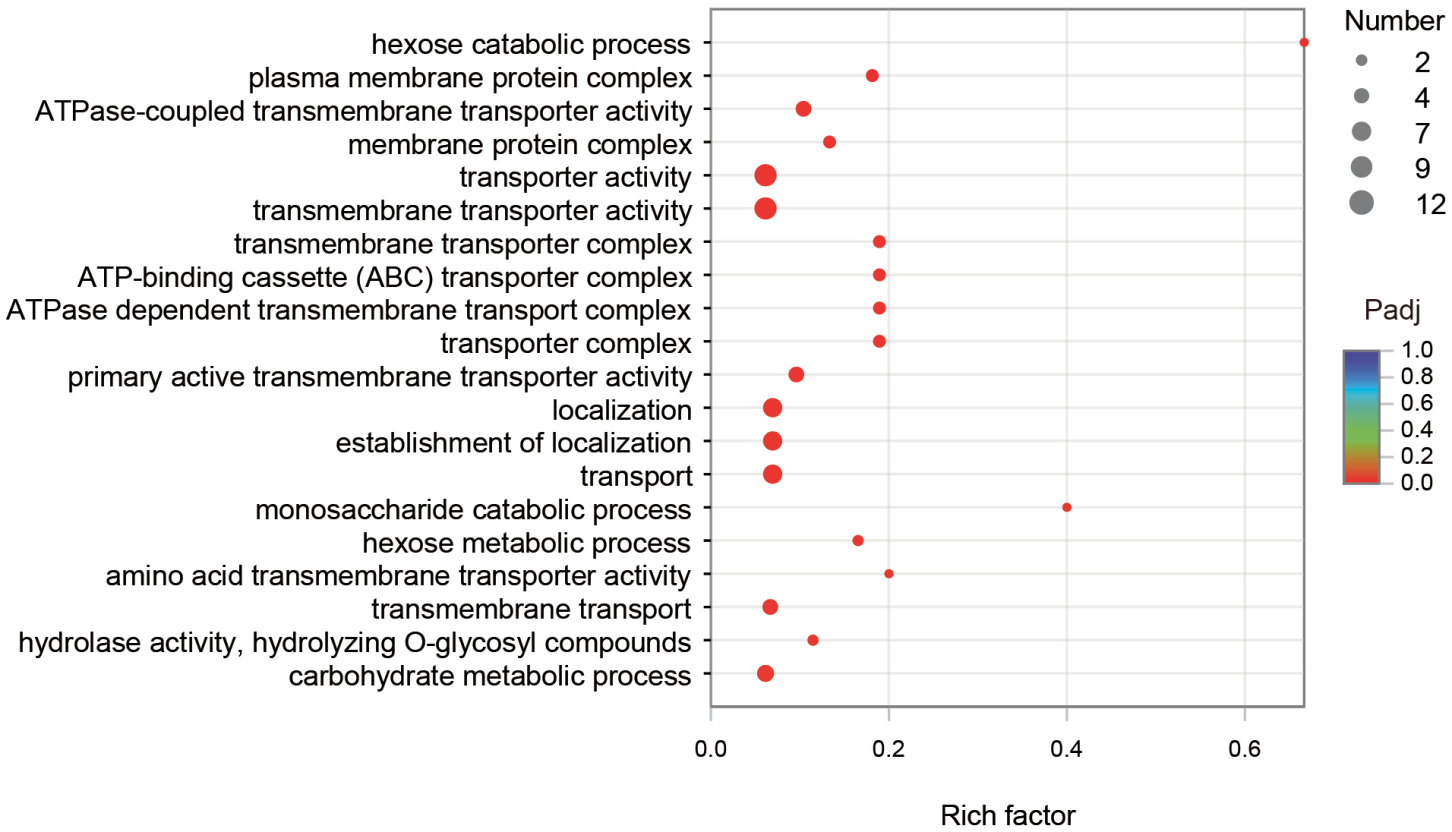
The GO enrichment of the SDE genes.

The GO enrichment of SDE genes showed that 53 terms were significantly enriched (Padj≤0.05), and most of the GO terms were involved in transport and carbohydrate metabolism functions (Figure 8). The enriched GO terms contained 29 SDE genes, including two glycoside hydrolase genes (1940 and 3616) and two ester linkage hydrolase genes (0600 and 1935). There was also one rhamnosidase gene (1947), three β-glucosidase genes (1932, 1941, and 1944), and one acetylesterase gene (1936) in the whole significantly upregulated genes. Therefore, it is speculated that encoding proteins of 0600, 1935, and 1936 genes may be involved in the hydrolysis of β-glucosyl ester linkage at C19 of RC. The encoding proteins of 1932, 1940, 1941, 1944, and 3616 genes may be involved in the hydrolysis of β-glucosidic linkages at C13 of RC. The encoding protein of gene 1947 may be involved in the hydrolysis of α-rhamnosidic linkage at C13 of RC.

### 3.5. Significantly differentially metabolic pathways in the RC metabolism

The KEGG enrichment of SDE genes showed that 7 pathways were significantly enriched (Figure 7). Most of the genes in map00940 and map00460 are missing in the genome, and these metabolic capabilities may not be present in CR5301. The map00500, map00040, and map00051 pathways are involved in the carbohydrate metabolism; the map02010 pathway is related to the transmembrane transport of metabolites; and the map00910 pathway is related to the synthesis of glutamic acid.

In CR5301, four genes (1932, 1940, 1941, and 1944) in the starch and sucrose metabolic pathway were significantly upregulated, and two genes (0917 and 1378) were significantly downregulated. All four genes (1932, 1940, 1941, and 1944) encode β-glucosidase [EC 3.2.1.21], but their upstream and downstream genes are either missing in the genome or downregulated in expression. These results indicated that the significant upregulation of transcription or expression of these four genes was not caused by sucrose metabolism but by RC metabolism. The gene 0917 encodes β-fructofuranosidase [EC 3.2.1.26], and the gene 1378 encodes levansucrase [EC 2.4.1.10]. These two genes are important in sucrose catabolism (Braga et al., 2022), and their downstream genes are either downregulated or of very low abundance. These results indicated that the transcription of 0917 and 1378 genes was significantly downregulated due to the decreased activity of catabolic sucrose in CR5301, caused by the addition of RC.

**Figure 9:**
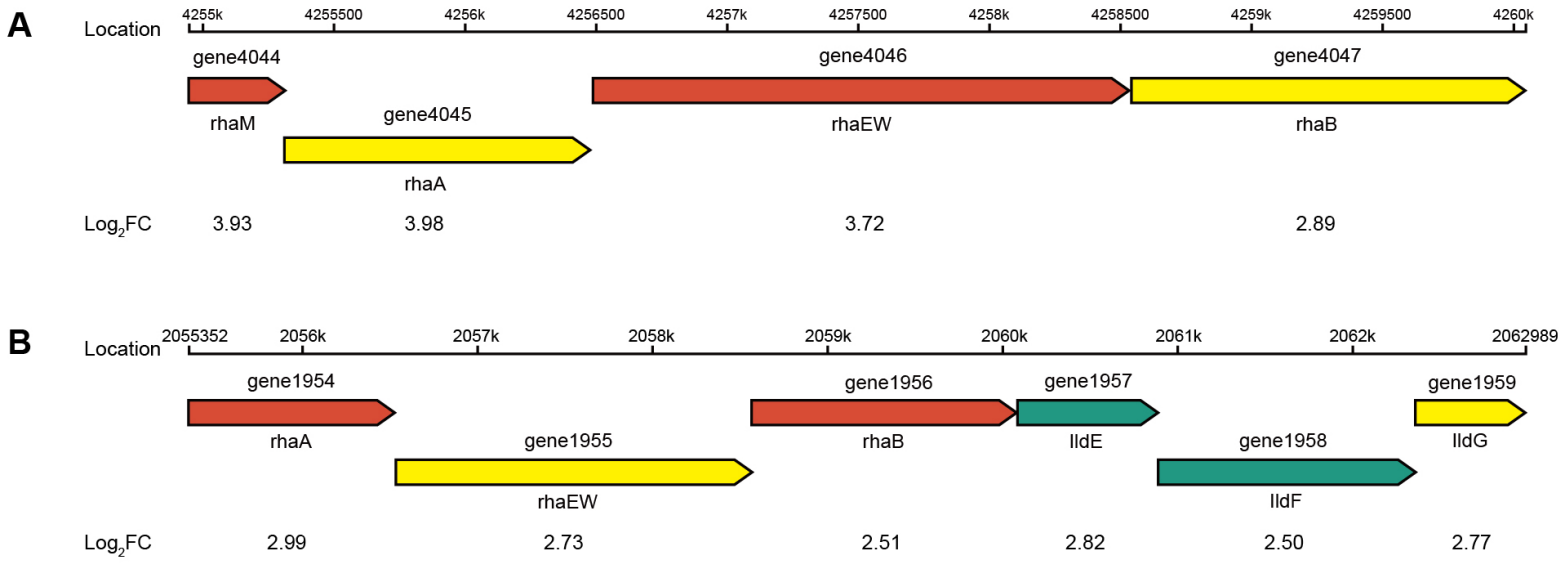
Putative genetic organization of the *rha* operons. **(A)** Rhamnose-degrading operon 1 and **(B)** rhamnose-degrading operon 2.

The four genes (1954, 1956, 4045, and 4047) were all significantly upregulated in the pentose and glucuronate interconversions pathway (map00040) and the fructose and mannose metabolism pathway (map00051) in CR5301. The gene 1377 was significantly downregulated in the map00051 pathway. The upstream and downstream genes of the four genes in these two pathways are all missing in the genome of CR5301. These results indicated that the significant upregulation of transcription or expression of these four genes was not due to the increased metabolism of map00040 and map00051 in host cells but rather in response to the added substrate RC. The genes 1954 and 4045 encode L-rhamnose isomerase [EC 5.3.1.14], and the genes 1956 and 4047 encode rhamnulokinase [EC 2.7.1.5]. They are both key enzymes in the bacterial rhamnose catabolic pathway via phosphorylation (Hirooka et al., 2016). At the same time, similar to the *rha*A and *rha*B genes previously identified, they form two similar structural rhamnose-catabolic operons with upstream and downstream genes (Hirooka et al., 2016). In addition, the expression of all genes in the two operons (one operon consisting of genes 4044–4047 and the other operon containing genes 1954–1959) was all significantly upregulated (Figure 9). These results further suggest that the upregulation of transcription/expression of these four genes and their operon is not related to map00040 and map00051 metabolism but is in response to rhamnose, which is hydrolyzed from RC. The gene 1377, encoding fructan β-fructosidase [EC 3.2.1.80], hydrolyzes inulin, levan, and sucrose. Therefore, similar to genes 0917 and 1378, the significant downregulation of 1377 was due to the relative decline in sucrose catabolic activity of CR5301 when RC was added to the medium.

In CR5301, three genes (0187, 3785, and 3786) were significantly upregulated, and six genes (0914, 0915, 0916, 2403, 2404, and 3149) were significantly downregulated in the ABC transporter pathway (map02010). The gene 0186–0189 encoding the phosphoric acid transport system (0187–0189 forms the operon) was upregulated, indicating that phosphate transport was upregulated, that is, CR5301 requires more phosphoric acid. The genes 3785 and 3786 (which form operons) encode multidrug efflux pumps and are significantly upregulated, indicating that some metabolites need to be effluxed. It is speculated that STE, the final product of RC metabolism, may be effluxed through this system. The genes 0902, 0913, 0914, 0915, 0916, and 0917 were downregulated or significantly downregulated. The Raffinose/Stachyose/Melibiose transportation system was encoded by the 0902, 0914, 0915, and 0916 genes, and the genes 0913 to 0917 formed an operon. Gene 0917 encodes β-fructofuranosidase. Therefore, these genes may be involved in sucrose transport and catabolism, and their downregulation may be a response to the decrease in sucrose catabolism. Genes 2403, 2404, and 2405 form an operon encoding biotin transport system; genes 3148, 3149, 3150, 3151, and 3152 form an operon encoding glutamate transport system. These genes are uniformly downregulated, indicating that biotin and glutamate transport are downregulated, suggesting that biotin and glutamate anabolic metabolism levels in CR5301 are reduced.

In the nitrogen metabolic pathway (map00910), six genes (1374, 1383, 1384, 1385, 1725, and 3744) were significantly downregulated, and other genes (1835, 1836, 3042, 3191, and 3631) in this pathway were also downregulated. These results indicated that the level of glutamate anabolic metabolism in CR5301 with nitrate as a substrate decreased significantly.

### 3.6. RT-qPCR verification

**Figure 10:**
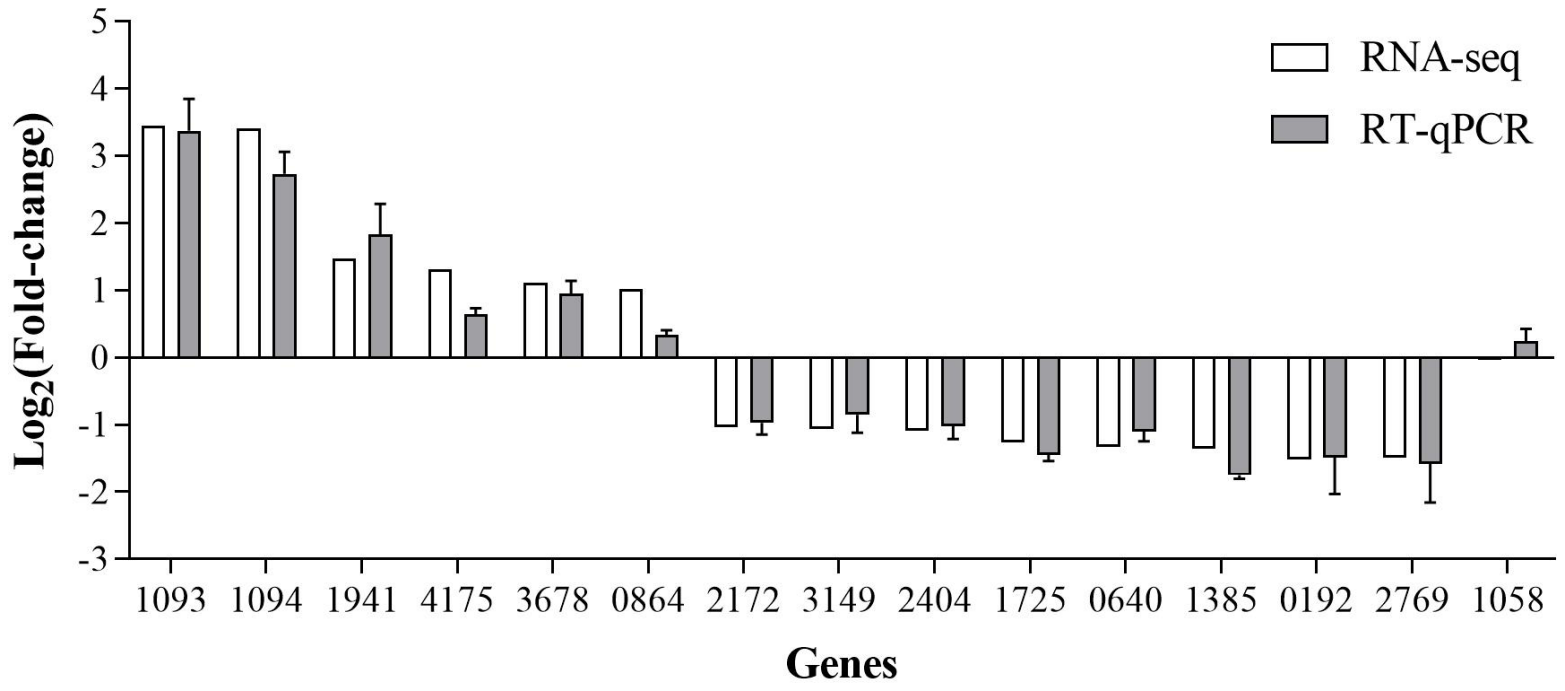
RT-qPCR verification of the SDE genes produced by RNA-seq.

The RT-qPCR results showed that the direction and trend of differential expression of all genes were consistent with that of RNA-seq (Figure 10). These results prove that the RNA-seq results are accurate and reliable.

## 4. Discussion

### 4.1. The catabolic pathway and mechanism of RC in CR5301

**Figure 11:**
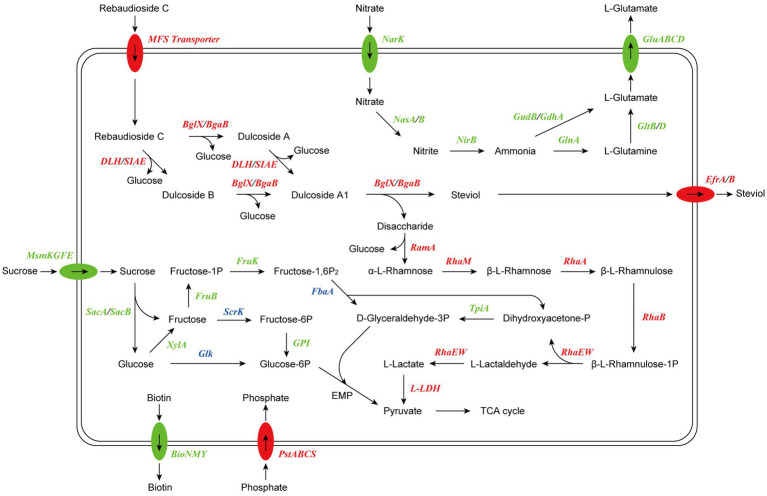
Proposed model of RC catabolism in CR5301. The abbreviations and corresponding encoding genes are listed in Supplementary Table S3. The enzymes marked in red are related to upregulated genes, the enzymes marked in green are related to downregulated genes, and the enzymes marked in blue are related to both upregulated and downregulated genes.

Based on the results of RNA-seq and metabolite identification, we proposed the complete pathway of RC catabolism in *P. ilicis* CR5301 under Czapek-Dox broth culture conditions (Figure 11). The pathway consists of two parts, namely, the hydrolysis of glycosyl side chains of RC and the degradation of hydrolyzed sugars.

The hydrolysis of glycosyl side chains is the primary task of microbial utilization of RC. *P. ilicis* CR5301 can efficiently hydrolyze RC because of its powerful glycoside hydrolases (70 genes) and carbohydrate esterases (37 genes) (Li et al., 2022a), which were associated with the hydrolysis of residues at the C13 and C19 sites in RC. The identification results of the metabolites showed that the hydrolysis of RC to STE involved two hydrolysis pathways (Figure 3), namely, RC-Duc A-Duc A1-STE and RC-Duc B-Duc A1-STE, both of which involved three-step enzymatic reactions corresponding to the hydrolysis of three glycosidic bonds in RC except for the α-1,2-rhamnosidic linkage. Therefore, we predict that the hydrolysis of glycosyl side chains of RC requires multiple enzymes to catalyze, which also implies that microorganisms containing single or few carbohydrate-active enzymes cannot or inefficiently hydrolyze RC to STE.

The metabolism of rhamnose is another necessary condition for microbial utilization of RC. The RNA-seq results showed that the gene 1947 of CR5301, which encodes a α-rhamnosidase, was significantly upregulated. Since no metabolites of the hydrolyzed α-1,2-rhamnosidic linkage at the C13 site were found, we predicted that the α-rhamnosidase could not directly hydrolyze α-1,2-rhamnosidic linkage at C13; however, we also predicted that other glycosidases would hydrolyze the glucosidic linkage in the disaccharide (α-L-rhamnopyranosyl-(1 → 2)-β-D-glucopyranose) chain at C13 of Duc A1 (Figure 3), and then, the disaccharide was hydrolyzed by this α-rhamnosidase to release monosaccharide rhamnose. Meanwhile, RNA-seq showed that CR5301 contained two *rha* operons, both of which were significantly upregulated (Figure 9), highly consistent with the pathway of rhamnose phosphorylation in known bacteria (Hirooka et al., 2016), and the second operon (Figure 9B) directly integrated three lactate dehydrogenase genes, which greatly improved the efficiency of rhamnose -utilization at the genetic level.

Previous studies have found that RC hydrolysis by fungi can occur either intracellularly or extracellularly (Ma, 2014), and the report on the conversion of RC by the bacterium *M. barkeri* XJ did not indicate cell localization (Jiang et al., 2020). Based on the results of RNA-seq analysis, we suggest that the RC catabolism in bacterium CR5301 probably occurred intracellularly due to two reasons: (i) there were no signal peptides in the esterase genes (0600, 1935, and 1936) and glycosidase genes (1932, 1940, 1941, 1944, and 3616), and only the gene 0600 had a transmembrane domain; thus, it was speculated that these enzymes were not secreted proteins and (ii) putative RC transporters MFS (genes 1877 and 1931) and STE efflux proteins EfrA/B (genes 3678, 3679, 3785, and 3786) were found by the SDE gene and KEGG enrichment analysis.

In conclusion, the above analysis results comprehensively revealed the detailed process, complete pathway, and cell localization of RC catabolism in strain CR5301. For the first time, the mechanism of RC catabolism in bacteria is clearly elucidated, which is characterized by two distinct stages, namely, the hydrolysis of glycosyl side chains of RC and the degradation of hydrolyzed oligosaccharides. These results expand the understanding of the catabolism of stevia glycosides in microorganisms and provide an important theoretical basis and potential enzyme resources for the microbial transformation and enzymatic modification of these glycosides with vital functional activities and a high value in the future.

### 4.2. The key enzymes of RC catabolism in CR5301

The identification results of the metabolites of RC showed that CR5301 could not completely degrade RC and only use the glycosyl groups in the side chain of RC as a carbon source, which was consistent with previous results observed in most organisms (Ma, 2014; Ma et al., 2014; Jiang et al., 2020). Therefore, enzymes that hydrolyze the glycosyl side chains of RC are the key enzymes in RC catabolism. The results of the SDE gene (the “SDE genes in the RC metabolism” section) indicated that three esterase genes (0600, 1935, and 1936) and five glycoside hydrolase genes (1932, 1940, 1941, 1944, and 3616) that were significantly upregulated may be involved in the hydrolysis of glycosyl side chains of RC. In other words, the transcriptional level evidence indicated that these genes may be the key genes in the catabolism of RC in the strain CR5301.

The hydrolysis of steviol glycosides by purified lipase has not been reported in previous studies. Only Milagre et al. (2009) studied the hydrolysis of ST by pancreatic, pancreatic lipase, and fungal lipase and found that these enzymes could hydrolyze ST to STE or isosteviol, but the hydrolysis efficiency was low. These results indicate that some lipases have the potential to hydrolyze the glycosyl side chains at the C13 and C19 sites in RC. Based on sequence homology, gene 0600 encodes dienelactone hydrolase, and genes 1935 and 1936 encode 9-O-acetylesterase. The dienelactone hydrolase and 9-O-acetylesterase have not been reported to have glucosidic and glucosyl ester linkage hydrolytic activities. Hence, whether they can hydrolyze RC needs to be further verified.

Previous studies on the hydrolysis and modification of steviol glycosides mainly focused on ST and RA, while there were few reports on RC. It has been reported that some β-glucosidase and β-galactosidase from microorganisms hydrolyze ST and RA to STE (Nguyen et al., 2019), demonstrating their ability to hydrolyze the β-glucosidic linkage of the C13 side chain and β-glucosyl ester linkage of C19 side chain (Chen et al., 2014; Nguyen et al., 2016), although the substrate specificity of enzymes from different sources varies greatly (Lan et al., 2019; Yan et al., 2021). Based on sequence homology, the proteins encoded by 1932, 1940, 1941, and 1944 genes belong to β-glucosidase of glycoside hydrolase family 3. The function and structure of β-glucosidase derived from many microorganisms have been characterized. Asp and Glu are catalytic nucleophiles and proton donors, respectively, and are essential hydrolytic active sites for β-glucosidase (Varghese et al., 1999). Multiple sequence alignment results showed that proteins encoded by 1932, 1940, and 1944 genes had the same amino acid residues of catalytic active and similar domains as known β-glucosidases (results not listed). Conversely, the protein encoded by the 1941 gene was missing Asp and Glu and was significantly smaller than known β-glucosidases (results not listed). Therefore, we predict that the protein encoded by gene 1941 may not be active for RC hydrolysis.

Gene 3616 encodes β-galactosidase belonging to glycoside hydrolase family 42, whose catalytic nucleophile and proton donor are both Glu (Shaikh et al., 2007; Lauro et al., 2008). Multiple sequence alignments showed that this protein had the same amino acid residues for catalytically active (localized to E160 and E318 in the protein encoded by gene 3616) and similar domains (results not listed) as known β-galactosidase.

Altogether, in addition to gene 1941, the esterase genes (0600, 1935, and 1936) and glycoside hydrolase genes (1932, 1940, 1944, and 3616) may be the key genes in the hydrolysis of RC in CR5301, which was further supported by literature and sequence analysis results. Moreover, the upregulation multiple, and abundance of the gene 1944 were the highest among these genes (Supplementary Table S2). Hence, we believe that the gene 1944 may play a central role in the hydrolysis of RC.

## Data availability statement

The original contributions presented in the study are included in the article/supplementary material, further inquiries can be directed to the corresponding author.

## Author contributions

HL wrote the manuscript and performed the experiment. DS edited the original manuscript and contributed significantly to the analysis. LC contributed to the conception of the study. BW helped to write the abstract and perform the analysis with constructive discussions. All authors contributed to the article and approved the submitted version.

## Funding

This study was supported by the University Nursing Program for Young Scholars with Creative Talents in Heilongjiang Province (UNPYSCT-2017112) and Heilongjiang Bayi Agricultural University Support Program for San Heng San Zong (ZRCPY202228), and the Doctor Research Startup Program of Heilongjiang Bayi Agricultural University (XDB202009)

## Conflict of interest

The authors declare that the research was conducted in the absence of any commercial or financial relationships that could be construed as a potential conflict of interest.

## Publisher’s note

All claims expressed in this article are solely those of the authors and do not necessarily represent those of their affiliated organizations, or those of the publisher, the editors and the reviewers. Any product that may be evaluated in this article, or claim that may be made by its manufacturer, is not guaranteed or endorsed by the publisher.
